# Time-Restricted Feeding Restores Obesity-Induced Alteration in Adipose Tissue Immune Cell Phenotype

**DOI:** 10.3390/nu13113780

**Published:** 2021-10-25

**Authors:** Youngyoon Lee, Yelim Kim, Minam Lee, Dayong Wu, Munkyong Pae

**Affiliations:** 1 Department of Food and Nutrition, Chungbuk National University, Chungdae-ro 1, Seowon-gu, Cheongju 28644, Korea; eowjs010@naver.com (Y.L.); yr097@naver.com (Y.K.); nam6658@gmail.com (M.L.); 2 Nutritional Immunology Laboratory, Jean Mayer USDA Human Nutrition Research Center on Aging at Tufts University, 711 Washington Street, Boston, MA 02111, USA; dayong.wu@tufts.edu

**Keywords:** insulin resistance, macrophages, obesity, T cells, time-restricted feeding

## Abstract

Studies suggest that time-restricted feeding (TRF) may prevent obesity and its commodities. At present, little is known about how TRF impacts immune cells, and whether such an effect is linked to altered metabolic parameters under condition of a high-fat diet (HFD)-induced obesity. To address these issues, we conducted a study in which we determined whether TRF has therapeutic efficacy against weight gain, adiposity, as well as associated immune cell disturbance found in obese mice. Six-week-old male C57BL/6 mice were fed a low-fat diet (LFD) or HFD ad libitum for six weeks, after which time a subgroup of HFD mice was switched to the 10 h TRF paradigm (HFD-TRF) for additional eight weeks. We found that TRF intervention reduced HFD-induced weight gain. Even with comparable fat mass and mean adipocyte area, the HFD-TRF group had lower mRNA levels of proinflammatory cytokine *Tnfα* and chemokine *Ccl8*, along with reduced numbers of adipose tissue macrophages (ATM), CD11c^+^ ATM, and CD8^+^ T cell compared to the HFD group, while maintaining CD8^+^ to CD4^+^ ratio at levels similar to those in the LFD group. Furthermore, TRF intervention was effective in improving glucose tolerance and reducing HOMA-IR. Taken together, our findings suggest that TRF restores the obesity-induced alteration in immune cell composition, and this effect may in part contribute to health benefits (including insulin sensitivity) of practicing TRF.

## 1. Introduction

Time-restricted feeding (TRF) is a particular form of intermittent fasting, which is mainly characterized by limiting the time window of energy intake within a few hours every day without an overt attempt at modification [1]. Compared with those with ad libitum access to a high-fat diet, mice that were subjected to daily TRF (8–15 h) during the active phase are largely protected from excessive body weight gain, adiposity, and/or insulin resistance under high-fat diet feeding and other nutritional challenges [2,3,4,5]. In animal studies, mice with pre-existing obesity due to ad libitum feeding of high-fat diet benefited from the therapeutic effects of TRF [2]. Similarly, human studies have shown that men with prediabetes exhibited improved insulin sensitivity and blood pressure after practicing TRF [6]. On the other hand, however, TRF out of sync with the circadian timing (i.e., feeding during the inactive phase) has been reported to be associated with negative impact on health, such as worsened glucose intolerance [7,8].

Obesity has been linked to the alteration in immune systems. Obese mice exhibited impaired T cell response to infection [9]. Diminished production of antibodies after vaccination was observed in obese individuals [10]. Studies in rodents and humans further elucidated that body fat mass is associated with changes in immune cell composition which is often characterized by a proinflammatory phenotype, such as increased white blood cells, neutrophils and proinflammatory monocytes in circulation [11,12]. In obesity, monocytes migrate more rapidly and to a greater extent into adipose tissue (AT) to become adipose tissue macrophages (ATM) [13]. While obesity increases ATM numbers, deletion of monocyte chemoattractant protein (MCP)-1 reduced macrophage infiltration into AT, together with improvement of diet-induced insulin resistance [14]. Of note, obesity dramatically increased CD11c^+^ ATM, which are highly enriched in the crown-like structures surrounding dead/dying adipocytes [15]. Depletion of CD11c^+^ cells resulted in rapid normalization of insulin sensitivity [16]. In addition to ATM, obesity increases the number of T cells present in AT [17,18], more toward CD8^+^ than CD4^+^ T cells [18]. CD8 depletion using neutralizing antibody was shown to ameliorate pre-established obese adipose inflammation and glucose intolerance [18]. These results indicate that obesity modulates immune cell phenotype, which in turn contributes to the pathogenesis of obesity-related insulin resistance.

While metabolic benefits of TRF have been reported in various studies, information is very limited concerning how TRF impacts immune cell phenotype. Complete blood counts indicated that total counts of white blood cells were decreased in peripheral blood of healthy participants during Ramadan but still within the normal range [19]. Similar observation was reported in those undergoing a fast twice a week during the daytime and eat at night [20]. Animal studies showed that a periodic fasting in aged mice could induce hematopoietic stem cells in bone marrow and reverse the age-dependent decline in the lymphoid to myeloid ratio in circulation [21]. Although different fasting protocols were used in those studies with the varied amount of calories consumed during the intervention period, these results do suggest that fasting may impact immune cell population. However, it is worth specifically verifying such an effect of TRF under the condition involving metabolic stressors such as a high-fat diet.

The aim of this study was to investigate the therapeutic effects of TRF in high-fat diet-induced overweight and obese mice. We evaluated whether TRF affects immune cell populations and inflammatory markers in AT, along with changes in body weight, adiposity, and glucose tolerance.

## 2. Subjects and Methods

### 2.1. Animals

All animal experiments were reviewed and approved by the Institutional Animal Care and Use Committee of Chungbuk National University (approval number: CBNUA-1347-20-01). Five-week-old male C57BL/6J mice were purchased from DBL (Eumsung, Korea) and housed (three to four mice per cage) under controlled temperature (23 °C ± 1 °C), relative humidity (50% ± 10%) and light/dark cycle (12-h dark/12-h light 7:00 a.m.–7:00 p.m.). After 1 week of adaptation, mice were then randomly divided into 2 groups and ad libitum fed the low-fat diet (LFD; 10% calories from fat, D12450B; Research Diets, Inc., New Brunswick, NJ, USA, *n* = 7) or high-fat diet (HFD; 60% calories from fat, D12492; Research Diets, Inc., New Brunswick, NJ, USA, *n* = 14). After 6 weeks of being fed experimental diets, the mice fed HFD were further divided into 2 groups (7 mice/group) to continue having ad libitum access to HFD or to have time-restricted access to food (HFD-TRF) for 8 more weeks. Under TRF, mice were allowed access to food for 10 h between ZT13 (1 h after lights off) and ZT23 (1 h before lights on). Food access was regulated by transferring mice daily between cages with food and water and cages with water only. To control for mouse handling, ad libitum-fed mice were also transferred between feeding cages at the same time. Food intake was measured twice a week and body weight was measured once a week. At the end of feeding period, mice were euthanized after 9 h of fasting (ZT22-ZT7). Blood was collected through cardiac puncture. AT, including epididymal, inguinal subcutaneous, retroperitoneal fat, was collected and weighed. A halved piece of the left epididymal fat pad was used for histological analysis and the remaining left piece was snap-frozen and stored at −70 °C for RNA analysis. The right epididymal fat pad was used for flow cytometry analysis.

### 2.2. Glucose Tolerance Test and HOMA-IR

A glucose tolerance test was performed two weeks prior to sacrifice after 6 h fasting period starting from ZT1. Blood was collected from tail veins of unanesthetized mice to measure glucose (Contour; Bayer) and serum insulin (Crystal Chem, Downers Grove, IL, USA). The formula for the homeostatic model of insulin resistance (HOMA-IR) was calculated as fasting blood glucose (mmol/L) × fasting insulin (mU/L)/22.5 [22]. After collecting baseline blood, mice were injected i.p. with 1.2 g glucose/kg of body weight, and blood glucose levels were measured at 15, 30, 60, and 120 min post-injection.

### 2.3. Histological Analysis

Epididymal fat pads were fixed in 4% formaldehyde (Sigma, St. Louis, MO, USA) overnight, embedded in paraffin, sectioned, and stained with hematoxylin and eosin. Digital images were acquired with a Leica DM 4000B microscope (Wetzlar, Germany). The size of the adipocyte area was determined using Image J software (National Institutes of Health, Bethesda, MD, USA).

### 2.4. Isolation of Stromal Vascular Fraction

Stromal vascular fractions (SVF) of AT were isolated by using a well-established collagenase-based method [23]. Briefly, epididymal fat pads were excised and minced into Krebs-Ringer bicarbonate (KRB) solution [24] followed by digestion with collagenase type II (1 mg/mL, Worthington, Lakewood, NJ, USA) at 37 °C for 20 min with shaking. The solution containing digested AT was filtered through a 250-μm strainer and centrifuged (300× *g*, 5 min) to separate floating adipocytes from the SVF pellet. Floating adipocytes were washed with KRB solution with EDTA (5 mmol/L, Invitrogen, Grand Island, NY, USA) and centrifuged again to separate residual SVF. Both SVF pellets were pooled and treated with ACK lysing buffer (Invitrogen) to remove red blood cells.

### 2.5. Flow Cytometry

SVF pellets were washed with PBS/2% FBS, incubated with Fc Block (BD Bioscience, San Jose, CA, USA) for 15 min and then stained with the following conjugated antibodies (30 min at 4 °C in the dark): CD45-APC-Cy7, CD3-APC, CD19-APC, NK1.1-APC, F4/80-PE-Cy7, CD11b-PerCP-Cy5.5 (all from BD Bioscience), and TER119-APC and CD11c-PE (eBioscience, San Diego, CA, USA) for ATM, CD45-APC-Cy7, CD19-PE, CD4-V500, CD8a-PerCP-Cy5.5, Gr1-APC (BD Bioscience), and CD3-eFlour450, NK1.1-PE-Cy7, TER119-APC, F4/80-APC (eBioscience) for lymphocyte population. After incubation, cells were washed and resuspended in PBS/2% FBS and then analyzed by FACS Canto II (BD Biosciences) and FlowJo software (version 10, Tree Star Inc., Ashland, OR, USA). As described previously [25], ATM were defined as CD45^+^, CD3^−^, CD19^−^, NK1.1^−^, TER119^−^, CD11b^+^ and F4/80^+^. T cells (CD19^−^CD3^+^NK1.1^−^), B cells (CD19^+^CD3^−^), and NK cells (CD19^−^CD3^−^NK1.1^+^) were gated after excluding the myeloid lineage (Gr1^+^, F4/80^+^) and red blood cells (TER119^+^) among immune cells (CD45^+^).

### 2.6. Quantitative Real-Time RT-PCR

Total RNA from frozen, pulverized epididymal fat was prepared by using an RNeasy Lipid Mini Kit (Qiagen, Valencia, CA, USA) according to the manufacturer’s protocol. One microgram of total RNA was reverse-transcribed into cDNA with an Advantage RT-for-PCR kit (Clonetech, Palo Alto, CA, USA). Taqman quantitative real-time RT-PCR was conducted using QuantStudio 5 Real-Time PCR system (Thermo Fisher Scientific, Waltham, MA, USA). Fold changes were calculated as 2^−^^ΔΔCt^ compared with the endogenous control gene, TATA box binding protein (TBP) using LFD as the reference group. The primers used in this study include *Adgre1* (Mm00802529_m1), *Itgax* (Mm00498698_m1), *Tnf* (Mm00443258_m1), *Ccl2* (Mm00441242_m1), *Ccl8* (Mm01297183_m1) and *Tbp* (Mm00446973_m1).

### 2.7. Statistics

Results were expressed as mean ± SEM. Data were analyzed by ANOVA followed by Tukey’s HSD *post hoc* procedure. Differences at *p* values smaller than 0.05 were considered significant. Statistical analysis was performed using SPSS software (version 25.0, SPSS, Chicago, IL, USA).

## 3. Results

### 3.1. Effects of Time-Restricted Feeding on Body Weight, Food Intake, and Energy Efficiency Ratio (EER)

**Figure 1:**
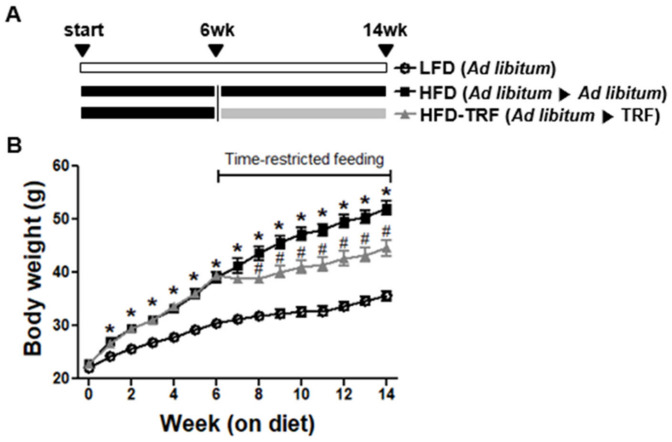
Therapeutic effects of time-restricted feeding (TRF) on body weight. (**A**) Schematic outline of the three feeding regimens used in this study. Six-weeks of C57BL/6J mice were fed low-fat diet (LFD) or high-fat diet (HFD) ad libitum for 6 weeks until 12 weeks of age. The HFD group were further divided two groups for the following regimens: continuous HFD ad libitum or HFD-TRF (10-h/daytime-restricted access to food during active period) for 8 weeks until 20 weeks of age. (**B**) Average body weight (±SEM, *n* = 7 mice). Significant differences among groups were determined by ANOVA followed by post hoc Tukey’s test. HFD, HFD-TRF (vs. LFD), * *p* < 0.05; HFD-TRF (vs. HFD), # *p* < 0.05.

As shown in Figure 1, mice fed a high-fat diet ad libitum (HFD group) gained more weight than those on a low-fat diet ad libitum (LFD group) (*p* < 0.05). At 6 weeks of feeding, when the HFD group were approximately 30% heavier than the LFD group (39.3 ± 0.38 g vs. 30.4 ± 0.57 g), the HFD group were randomly divided into 2 groups to continue HFD ad libitum and to start HFD-TRF with 10 h food access during the active phase, respectively. Two weeks after initiating the TRF program, the HFD-TRF group had significantly less weight gain than the HFD group (*p* < 0.05). During 8 weeks of intervention, the HFD-TRF group of mice exhibited a weight gain by 12.7%, which was significantly less than the weight gain by 33.5% seen in the HFD group (Table 1). At the end of experiment, the body weight of the HFD-TRF group were 16.8% lower than the HFD group. The average dietary intake in the HFD group was significantly higher than that in the HFD-TRF group. The energy efficiency ratio (EER) was used to assess the efficiency of animals in converting energy consumption into increased body weight. We found that EER was significantly higher in the HFD group compared to the LFD group, while mice in the HFD-TRF group exhibited EER values similar to those in the LFD group. These results indicate that TRF intervention may have metabolic benefits beyond controlling dietary intake (Table 1).

### 3.2. Effects of Time-Restricted Feeding on Adiposity and Adipose Tissue Inflammatory Infiltration

**Figure 2:**
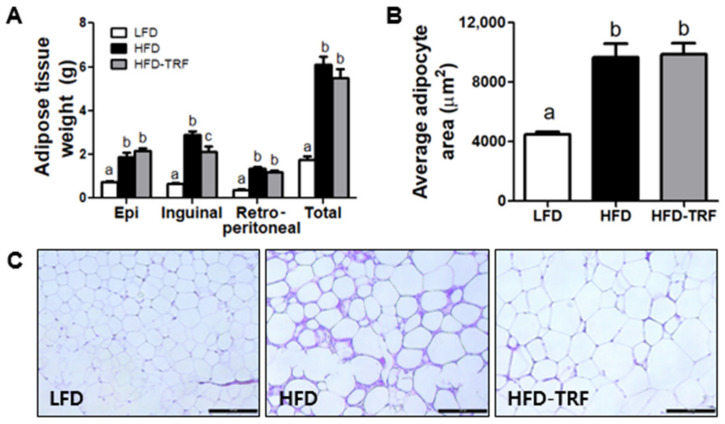
Effects of time-restricted feeding (TRF) on adipose tissue. C57BL/6 mice were fed a low-fat diet or high-fat diet ad libitum (LFD and HFD, respectively) for 14 weeks. Time-restricted feeding was started after 6 weeks of ad libitum consumption of a high-fat diet. (**A**) Fat distribution: epididymal adipose tissue, inguinal subcutaneous adipose tissue, retroperitoneal adipose tissue; (**B**) average adipocyte size area; (**C**) representative H&E-stained histological sections of epididymal adipose tissue in the LFD, HFD, and HFD-TRF groups. Scale bar, 100 μm. Data are presented as mean ± SEM (*n* = 7). ^a,b,c^ Different superscripts indicate significant difference at least at *p* < 0.05 by ANOVA with Tukey’s *post hoc* test.

There was no significant difference in epididymal and total fat mass between the HFD and HFD-TRF groups (Figure 2A). In addition, histologic examination of epididymal AT revealed that the mean adipocyte area was similar between the two groups. However, immune cell infiltration into AT was more pronounced in the HFD group compared to the HFD-TRF group (Figure 2B,C). These results indicate that TRF could reduce AT inflammation associated with obesogenic diets, even without a noticeable difference in adiposity.

### 3.3. Effects of Time-Restricted Feeding on ATM Content and Phenotype

**Figure 3:**
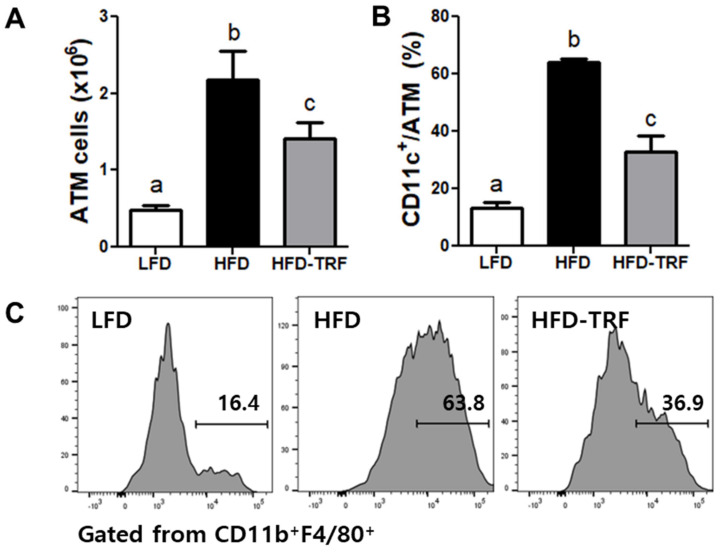
Effects of time-restricted feeding (TRF) on infiltration of adipose tissue macrophages (ATM). Epididymal adipose tissue was analyzed by flow cytometry after 14 weeks of different feeding regimens. LFD, low-fat diet ad libitum; HFD, high-fat diet ad libitum; HFD-TRF, 8 weeks of time-restricted feeding after 6 weeks of high-fat diet ad libitum. (**A**) Total ATM numbers; (**B**) frequency of CD11c^+^ ATM; (**C**) representative flow cytometry histograms of CD11c expression in ATM. Data are presented as mean ± SEM (*n* = 7). ^a,b,c^ Different superscripts indicate significant difference at least at *p* < 0.05 by ANOVA with Tukey’s *post hoc* test.

Flow cytometric analysis on the SVF of the epididymal fat pads revealed that ad libitum of a high-fat diet increased the number of total ATM by 3.5-fold compared to the LFD group, while TRF intervention halved this number (Figure 3A). In addition, we observed significant difference in ATM phenotype, i.e., more than 60% of ATM were CD11c^+^ in the HFD group relative to only 32.8% CD11c^+^ ATM in the HFD-TRF group (Figure 3B,C).

### 3.4. Effects of Time-Restricted Feeding on T Cell Accumulation in AT

**Figure 4:**
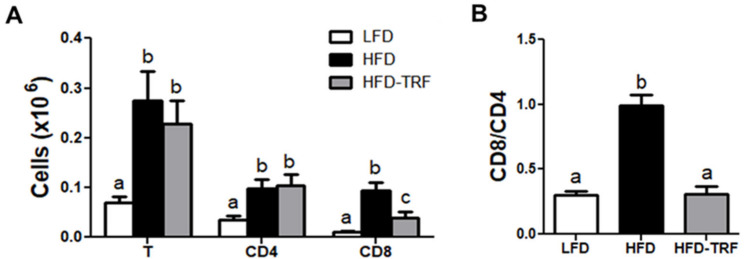
Effects of time-restricted feeding (TRF) on infiltration of T cells. Stromal vascular fraction of epididymal adipose tissue was analyzed by flow cytometry after 14 weeks of different feeding regimens. LFD, low-fat diet ad libitum; HFD, high-fat diet ad libitum; HFD-TRF, 8 weeks of time-restricted feeding after 6 weeks of high-fat diet ad libitum. (**A**) Total T, CD4 T, CD8 T cell numbers; (**B**) ratio of CD8 to CD4 T cells. Data are presented as mean ± SEM (*n* = 7). ^a,b,c^ Different superscripts indicate significant difference at least at *p* < 0.05 by ANOVA with Tukey’s *post hoc* test.

Compared with the LFD group, increased numbers of T cells, both CD4^+^ and CD8^+^ T cells, were found in AT of the HFD and HFD-TRF groups (Figure 4A). Interestingly, the HFD-TRF group had smaller number of CD8^+^ T cells but similar number of CD4^+^ T cells in AT relative to the HFD group, which resulted in the ratio of CD8^+^ to CD4^+^ T cells being restored to a level similar to that seen in the LFD group (Figure 4B). TRF intervention had no effect on the obesity-induced infiltration of B cells and NK cells in AT (Supplementary Figure S1).

### 3.5. Effects of Time-Restricted Feeding on Inflammatory Mediator Gene Expressions in AT

**Figure 5:**
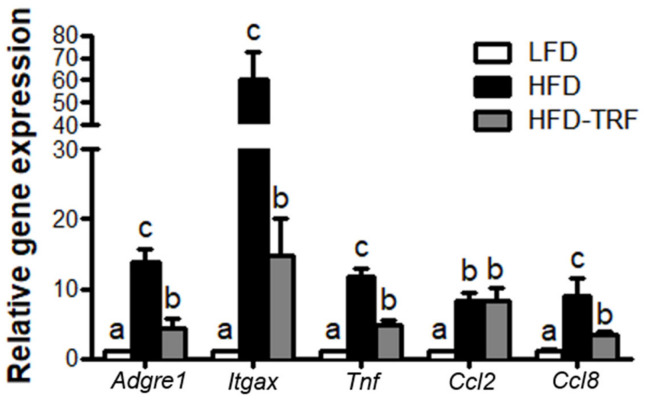
Effects of time-restricted feeding (TRF) on inflammatory mediator gene expressions in AT. Quantitative real-time RT-PCR analysis of selected proinflammatory macrophages (F4/80 and CD11c), cytokine (TNFα) and chemokines (Ccl2, Ccl8) in epididymal fat after 14 weeks of different feeding regimens. LFD, low-fat diet ad libitum; HFD, high-fat diet ad libitum; HFD-TRF, 8 weeks of time-restricted feeding after 6 weeks of high-fat diet ad libitum. Data are presented as mean ± SEM (*n* = 7). ^a,b,c^ Different superscripts indicate significant difference at least at *p* < 0.05 by ANOVA with Tukey’s *post hoc* test.

Real-time RT-PCR of the total AT for the macrophage-associated genes *Adgre1* (F4/80) and *Itgax* (CD11c) also indicated that HFD ad libitum increased proinflamamtory ATM infiltration (Figure 5), consistent with the flow cytometry data (Figure 3A,B). In addition, HFD ad libitum resulted in a significant elevation of *Tnf* (*TNFα*) and chemokine *Ccl8* (MCP2) mRNA levels in AT (11.8-fold, 9.0-fold, respectively), where TRF decreased them by ~60%.

### 3.6. Effects of Time-Restricted Feeding on Glucose Homeostasis

**Figure 6:**
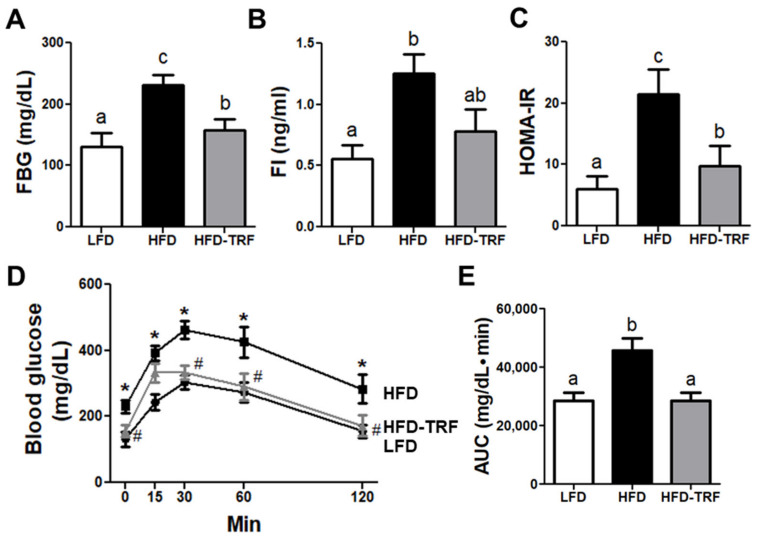
Effects of time-restricted feeding (TRF) on HOMA-IR and glucose tolerance. C57BL/6 mice were fed with a low-fat diet ad libitum (LFD), high-fat diet ad libitum (HFD), high-fat diet ad libitum followed by time-restricted feeding (HFD-TRF) for 12 weeks. (**A**) Fasting blood glucose; (**B**) fasting insulin; (**C**) insulin resistance index (HOMA-IR), measured as FBG (mmol/L) × FI (mU/L)/22.5. (**D**,**E**) Glucose and area of glucose under the curve (AUC) during glucose tolerance test upon i.p. injection of 1.2g glucose/kg body weight. Data are presented as mean ± SEM (*n* = 7). ^a,b,c^ Different superscripts indicate significant difference at least at *p* < 0.05 by ANOVA with Tukey’s *post hoc* test. HFD (vs. LFD), * *p* < 0.05; HFD-TRF (vs. HFD), ^#^ *p* < 0.05.

While ad libitum consumption of HFD elicited a significant elevation in fasting blood glucose (FBG), this adverse effect of HFD was largely alleviated by TRF (230.6 ± 17.7 vs. 157.4 ± 17.8 mg/dL in HFD ad libitum and HFD-TRF, respectively) to a level comparable to the LFD group (131.3 ± 22.3 mg/dL; Figure 6A). In addition, the HFD-TRF group tended to have lower fasting insulin (FI) levels compared with the HFD group (*p* = 0.068; Figure 6B). Consequently, the HFD-TRF group showed significantly lower HOMA-IR, an index of insulin sensitivity, compared with the HFD group (Figure 6C), suggesting that TRF attenuates insulin resistance associated with a high-fat diet. In line with these findings, following i.p. injection of glucose, mice in HFD-TRF exhibited an improvement of glucose tolerance (Figure 6D,E).

## 4. Discussion

In this study, we investigated the therapeutic effect of TRF on body weight and adiposity, as well as its impact on immune cell homeostasis disturbed during obesity in C57BL/6J mice. TRF intervention started after six weeks of HFD feeding attenuated weight gain. Although there was no statistical significance in fat mass and mean adipocyte area, the HFD-TRF group had greatly reduced the numbers of total ATM, proinflammatory CD11^+^ATM, and CD8^+^ T cells compared to the HFD group, while restoring the CD8^+^ to CD4^+^ ratio to a level similar to that in the LFD group. In addition, the HFD-TRF group exhibited reduced mRNA levels of *Tnfα*, a potent proinflamamtory cytokine produced by ATM [26], and monocyte chemoattractant *Ccl8* in AT. This decreased AT inflammation by TRF intervention is associated with, and may partially contribute to, the improvement in glucose tolerance.

Our results showed that 8 weeks of daily 10 h-TRF intervention in overweight/obese mice could reduce weight gain (12.7% vs. 33.5% increase in HFD ad libitum). Previous studies have shown that TRF for 8 to 15 h during active period can be a preventive measure against obesity in mice [2,3,4], although the reported efficacy varies depending on daily feeding window, e.g., a 26% gain for 9 h TRF, a 43% gain for 15 h TRF versus a 65% gain in mice fed ad libitum for 9 to 12 weeks [2]. Although we did not see the dramatic weight loss in our 10 h-TRF protocols in mice weighed around 40 g compared to 9 h-TRF in mice over 50 g [2], the increased energy efficiency ratio in HFD group was greatly reduced by TRF intervention. This reduced efficiency of animals in converting energy consumption into body weight could in part contribute to the reduced body weight of the HFD-TRF group. Previously, it has been shown that mice on HFD-TRF for 8 h have increased activity and energy expenditure toward the end of the night [2], and increased expression of uncoupling protein 1 to 3 during the late night correlates with increased energy expenditure in mice on HFD-TRF [3]. Thus, we believe that lower body weight associated with TRF regimen may be partly related to differences in energy expenditure and restricting food access window is helpful for weight management beyond controlling dietary intake.

In contrast to several previous studies that reported lower fat mass with TRF [27,28], we found that inflammatory cell infiltration was more prominent in the HFD group even with comparable adiposity compared to the HFD-TRF group. In addition, we noticed that dead adipocytes, detected as crown-like structures, were prevalent in visceral fat depots of the HFD group but almost absent in HFD-TRF mice. Thus, our results clearly suggest that TRF could help keep AT in check under the condition of HFD feeding.

The numbers of total ATM and proinflammatory ATM (defined as CD11c^+^ ATM) were higher in obese mice fed HFD ad libitum compared with mice fed LFD ad libitum as well as those fed HFD for restricted time (10 h/d) in our study. The increase in ATM, especially those with inflammatory properties, indicates increased AT inflammation. Indeed, we observed that HFD ad libitum upregulated mRNA expression of *Tnfα* in AT, a proinflammatory cytokine mainly expressed by ATM [26], where TRF reduced it by ~60%. Thus, TRF intervention-induced inhibition in infiltration of the immune cells into AT and gene expression of proinflammatory molecules represents an overall effectiveness of TRF’s protection against AT inflammation. Previously, TRF for 8 h per day was shown to reduce mRNA levels of *Tnfα, Cxcl2, Il6, Il1* in fat pads of C57BL/6 mice fed a high-fat diet [3]. Similarly, the decreased expressions of *Ccl8* and *Tnfα* were observed when transferring the mice from ad libitum to TRF of high-fat diet [2]. Jordan et al. [29] showed that a 20 h-fasting in mice reduced the number of monocytes in circulation and AT and repeated 24 h-fasting in mice for 4 weeks improved chronic inflammatory disease such as autoimmune encephalomyelitis. However, some other studies reported the results inconsistent with those mentioned above. For instance, Asterholm et al. [30] showed that mRNA expressions of macrophage marker F4/80 and putative M1 marker CD11c were not altered by a 24 h-fasting in AT of obese mice, and Kosteli et al. [31] reported that a 24 h-fasting increased mRNA expression and immunohistochemical staining of F4/80 in AT of obese mice. One of reasons for these discordant results is probably due to use of different fasting protocols (one-time fasting vs. daily TRF). While the duration of feeding and fasting may affect the magnitude of weight loss [2], our results clearly showed that daily 10 h-TRF reduced HFD-induced excess infiltration of inflamed macrophages into AT.

However, ATM is not only immune cells regulated by TRF intervention in AT. We found that HFD feeding increased accumulation of CD8^+^ T cells in obese epididymal fat pads leading to higher ratio of CD8^+^ to CD4^+^ T cells, which was normalized by TRF treatment. Increased ratio of CD8^+^ to CD4^+^ T cells is often viewed undesirable because it is often associated with the condition such as inflammation [32,33] and aging [34,35]. Thus, it appears that 10 h-TRF in the active phase may be effective in maintaining immune homeostasis within AT and possibly beyond, which needs further studies.

In this study, we found that 10 h-TRF during the active phase in mice fed HFD had lower insulin resistance index HOMA-IR than those fed HFD ad libitum. In addition, glucose tolerance test revealed that 10 h-TRF protected mice from HFD-induced impairment of glucose tolerance. Consistent with our results, other investigators reported that daily TRF (9–15 h) reduced insulin resistance and improved glucose tolerance in obese mice associated with obesogenic diets [2,5]. Adipocyte hypertrophy in diet-induced obesity is often accompanied by the accumulation of proinflammatory immune cells in AT. In the current study, however, even though the HFD-TRF group had average adipocyte areas similar to the HFD group, TRF was still effective in reducing the HFD-induced accumulation of ATM, CD11c^+^ ATM, and CD8^+^ T cells. In obese rodents and individuals, ATM accumulation is a critical component in the development of obesity-induced inflammation [26,36,37]. It is known that the recruited macrophages in AT express high levels of the inflammatory factors known to contribute to systemic inflammation and insulin resistance [36,38]. While resident ATM do not express CD11c, ATM that are newly recruited by increased adiposity express CD11c [13,39]. When CD11c^+^ cells were genetically deleted, HFD-induced inflammation, glucose tolerance, and insulin resistance were normalized in obese mice. Along with macrophages, obesity increased CD8^+^ T cell numbers in AT [18,40]. Depletion of CD8^+^ T cells was shown to improve obesity-induced insulin resistance, which is associated with a specific decrease in CD11c^+^ ATM numbers [18]. Thus, based on our results, reduction in CD11c^+^ ATM and CD8^+^ T cells present in AT may in part explain the protective effect of TRF intervention against glucose intolerance and insulin resistance.

One limitation of this study is that only changes in adipose tissue were analyzed but systemic (circulating) inflammation was not assessed. However, based on the information in the literature, we speculate that systemic inflammation status would have gone along as well because serum concentrations of TNF-α [41] and IL-6 [42] have been shown to be down-regulated by TRF intervention. Future work should explore systemic changes, other than fat, including both phenotype and functions of peripheral immune cells as well as hematopoiesis in bone marrow.

## 5. Conclusions

In summary, in this study, we showed that TRF intervention effectively reduced weight gain and energy efficiency (weight gain/caloric consumption) in overweight/obese mice. Although total fat mass and mean adipocyte area were comparable between the HFD and HFD-TRF, infiltration of CD11c^+^ macrophages and CD8^+^ T cells into AT were greatly decreased in the HFD-TRF group compared to HFD group. Concomitantly, we found a significant decrease in levels of insulin resistance index HOMA-IR and improvement in glucose tolerance test. Together these results suggest a great potential for using TRF regime to counteract obesity-induced inflammatory infiltration of immune cells in AT, which, in turn, may significantly help in fighting against systemic insulin resistance and glucose intolerance, and possibly also other related metabolic disorders.

## Figures and Tables

**Table 1:** Body weight, food intake, and energy efficiency ratio.

Measurements			Groups	
		LFD	HFD	HFD-TRF
Body weight (g)	6 weeks of age	22.10 ± 0.36 ^ns^	22.84 ± 0.21	22.87 ± 0.32
	12 weeks of age	30.40 ± 0.57 ^a^	39.00 ± 1.04 ^b^	39.53 ± 0.55 ^b^
	20 weeks of age	35.61 ± 0.90 ^a^	52.08 ± 1.33 ^c^	44.57 ± 1.53 ^b^
Food intake (g/d)		2.98 ± 0.07 ^c^	2.63 ± 0.04 ^b^	2.24 ± 0.05 ^a^
EER ^1^ (%)		0.83 ± 0.07 ^a^	1.71 ± 0.08 ^b^	0.77 ± 0.17 ^a^

Six-weeks of C57BL/6J mice were fed low-fat diet (LFD) or high-fat diet (HFD) ad libitum for 6 weeks until 12 weeks of age. The HFD group were further divided two groups for the following regimens: continuous HFD ad libitum or HFD-TRF (10-h/daytime-restricted access to food during active period) for 8 weeks until 20 weeks of age. ^1^ EER (Energy Efficiency Ratio) = body weight gain/caloric consumption during 8 weeks of intervention × 100. Data are presented as mean ± SEM (*n* = 7). ^a,b,c^ Different superscripts indicate significant difference at least at *p* < 0.05 by ANOVA with Tukey’s *post hoc* test. ns: non-significant.

## Data Availability

The data presented in this study are available on request from the corresponding author.

## Supplementary Materials

The following are available online at https://www.mdpi.com/article/10.3390/nu13113780/s1. Figure S1: Effects of time-restricted feeding (TRF) on infiltration of B cells and natural killer (NK) cells.
