# Supplementary material for: Time-Restricted Feeding Restores Obesity-Induced Alteration in Adipose Tissue Immune Cell Phenotype
Source: Nutrients. 2021 Oct 25;13(11):3780. doi: 10.3390/nu13113780 (PMC8623978; doi:10.3390/nu13113780)
Supplement: Supplementary file 1 [file nutrients-13-03780-s001.zip › nutrients-1418828-supplementary.pdf]

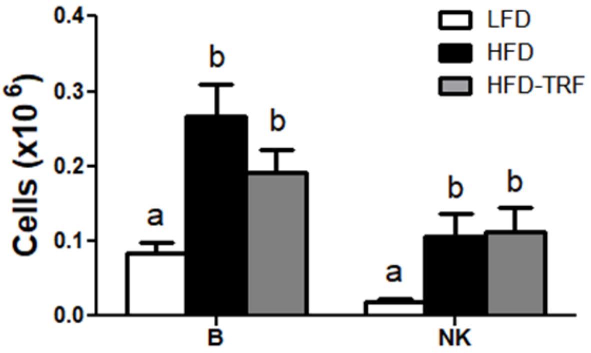

Supplementary Figure S1. Effects of time-restricted feeding (TRF) on infiltration of B cells and natural killer (NK) cells. Stromal vascular fraction of epididymal adipose tissue was analyzed by flow cytometry after 14 weeks of different feeding regimens. LFD, low-fat diet *ad libitum*; HFD, high-fat diet *ad libitum*; HFD-TRF, 8 weeks of time-restricted feeding after 6 weeks of high-fat diet *ad libitum*. Data are presented as mean ± SEM (n = 7). [a,b] Different superscipts indicate significant difference at least at $p < 0.05$ by ANOVA with Tukey's *post hoc* test.
